# Microplastics as active modulators of *Escherichia coli* biofilm characteristics and their implications on the development of antimicrobial resistance

**DOI:** 10.1016/j.bioflm.2026.100355

**Published:** 2026-02-11

**Authors:** Yanina Nahum, Neila Gross, Johnathan Muhvich, Muhammad H. Zaman

**Affiliations:** a Department of Biomedical Engineering, Boston University, Boston, MA, USA; b Center on Forced Displacement, Boston University, Boston, MA, USA; c Odum School of Ecology, University of Georgia, Athens, GA, USA; d Department of Materials Science and Engineering, Boston University, Boston, MA, USA

## Abstract

Microplastics are increasingly recognized as substrates that facilitate microbial colonization and may contribute to antimicrobial resistance, yet their role in shaping biofilm physiology remains poorly understood. Here, we investigated the antibiotic susceptibility, structural features, mechanical properties, and composition of extracellular polymeric substances (EPS) of *Escherichia coli* (*E. coli*) biofilms grown under flow and under identical conditions with three different materials: control (C), glass microbeads (G), and microplastic 10-μm beads (MP). We performed 24h antibiotic susceptibility tests using ciprofloxacin and found significantly enhanced tolerance in MP-biofilms, with approximately 60% of cells remaining viable after exposure to 350 μg/mL, compared to 24% in G-biofilms and minimal survival in controls at lower concentrations of ciprofloxacin (P < 0.0001). Reducing microplastic concentrations ten-fold did not enhance susceptibility, whereas lighter, hollow glass beads generated significantly more susceptible biofilms. MP-biofilms were shown to be nearly seven times thicker than control biofilms and exhibited localized zones of high cell density surrounding the microbeads. We further observed lower creep compliance in MP- and G-biofilms relative to controls, indicating increased stiffness. Finally, we analyzed EPS matrix composition and found that only MP-biofilms displayed substantial enrichment across all EPS components, especially proteins (>2.5-fold increase, P < 0.0001). Together, these results indicate that microplastics can not only serve as favorable surfaces for bacterial attachment and colonization but also actively promote biofilm architectures and biochemical features that confer elevated antibiotic tolerance. Our findings highlight microplastics as contributors to drug-tolerant biofilm microbial communities and reinforce their role as emerging environmental drivers of antimicrobial resistance.

## 1 Importance

Antimicrobial resistance is one of the globe's most pressing health problems. Environmental biofilms are an active driver of resistant pathogens in systems that people interact with frequently. This study shows that microplastics, a pervasive environmental contaminant found in all known ecosystems, are not passive surfaces but active modulators of biofilm physiology and antibiotic tolerance. These microplastic-associated biofilms incorporate more particles, develop localized high-density regions, and likely support enhanced cell–cell signaling and horizontal gene transfer, conditions that favor the accumulation and spread of antibiotic resistance genes. Because microplastics, antibiotics, and resistance genes frequently co-occur in wastewater and aquatic systems, our findings provide a mechanistic basis for viewing microplastics as environmental reservoirs and amplifiers of antimicrobial resistance. This work highlights the need to consider material properties and EPS-targeted strategies in environmental management and wastewater treatment to mitigate microplastic-facilitated antibiotic tolerance.

## 2 Introduction

Antimicrobial resistance (AMR) is a pressing global health crisis that is characterized by the ability of microorganisms to withstand the effects of antimicrobial agents, making antibiotics and other drugs inefficient [1,2]. In 2021, 4.71 million deaths involved drug-resistant infections, such as lower respiratory, bloodstream, and intra-abdominal infections, while 1.14 million of those deaths were directly attributable to AMR and could potentially have been prevented with effective antibiotics [3]. In addition, about 39 million more deaths are projected to occur between 2025 and 2050 as a result of AMR. While the AMR-human interface (i.e., overuse, inappropriate use, and uncontrolled discharge of antimicrobials, poor prescription patterns, etc.) has been well documented [[4], [5], [6]], research related to the impact of the environmental distribution of contaminants on AMR is still limited [7,8].

Wastewater systems, either from households, hospitals, farms or industries, have been identified as significant reservoirs of genetic material and resistant pathogens, and are therefore considered important contributors to AMR [9]. The presence of pollutants originating from industrial and agricultural runoff serves as a rich and diverse habitat for a wide combination of microorganisms that frequently organize into biofilms [10]. These highly organized structures are surrounded by a protective gel-like matrix of extracellular polymeric substances (EPS) that provides adhesion to surfaces, cohesion among bacteria, and protection against antimicrobials [11]. The close proximity of cells within biofilms facilitates intercellular communication and horizontal gene transfer, increasing the exchange of antibiotic-resistance genes and promoting resistance. For these reasons, biofilms are generally 100 to 1000 times more resistant than planktonic bacteriaa [12], and are therefore key drivers of AMR in wastewater systems [13].

Over the last decades, a dramatic increase in plastic use has made wastewater a significant repository for microplastics (MPs) [14,15]. These insoluble synthetic particles derive from global plastic production and are exacerbated by inadequate waste management, and have proved to be very difficult to control [16,17]. They can disperse into surface waters, accumulate in sediments, and persist through wastewater treatment processes, while interacting with surrounding organisms. Their unique physicochemical properties, such as hydrophobicity and surface roughness, distinguish them from other particles commonly found in wastewater, including glass, and make them exceptional substrates for biofilm formation [18,19], further enhancing cell-to-cell communication, the transfer of genetic material, and potentially contributing to enhanced AMR in wastewater systems.

In this study, we cultured biofilms under flow conditions in the presence of MPs or glass microbeads and compared them with biofilms grown without microbeads. Our culturing standards, including nutrient availability as well as flow conditions, were set to mimic realistic environmental settings, similar to those developed in wastewater. We performed antibiotic susceptibility tests for each of these biofilms. We then compared the physical properties, analyzed differences in EPS matrix composition, and explored their mechanical properties to understand possible mechanisms by which MPs may enhance resistance to antibiotics among other materials. This work provides novel insights into how the unique physicochemical properties of MPs influence biofilm physical and chemical characteristics and resilience, and offers mechanistic insights into their potential role in AMR in the environment. Given the widespread presence and persistence of MPs in wastewater and other aquatic environments and considering that biofilms are reservoirs of MPs, understanding their role in biofilm-mediated AMR is essential for informing environmental management and public health strategies.

## 3 Materials and methods

### 3.1 Strains, biofilm growth, and microbeads

Cultures of *E. coli* MG1655 that expressed GFP were used to grow biofilms for antibiotic susceptibility tests, structure and morphology, and microrheology. The wild-type *E. coli* WT-MG1655 (without GFP expression) was used to determine EPS matrix composition. Biofilms were grown in either 6-channel or 1-channel Ibidi® Ibitreat surface-treated flowcells (hydrophilic and slightly negatively charged surface, according to manufacturer) 0.4 mm or 0.6 mm deep, respectively (Ibidi Inc, USA) following existing protocols [20]. Briefly, liquid cultures were grown in Luria-Bertani (LB) broth overnight and incubated at 37 °C on a shaker. The cultures were then diluted to OD_600_ = 0.1 in LB broth, injected into the flow cells, and incubated under static conditions overnight. Biofilms were then grown under flow using 10% LB with a Golander peristaltic pump (BT100S, PH DG10-8, Golander, USA), at approximately 3 mL/h for 4 days or 2.5 mL/h for 3 days with single or 6-channel flowcells, respectively.

Polystyrene microbeads (8-12 μm diameter, density = 1.05 g/ml, 468312-100G, Millipore Sigma, Germany), soda-lime glass microspheres (8-12 μm diameter, density = 2.5 g/ml, P2015SL-2.5, Cospheric, USA), or hollow-glass microspheres (5-30 μm diameter, density = 0.6 g/ml,HGMS-0.6, Cospheric, USA) were added to the media at approximately 1 × 10^6^ microbeads/mL (unless otherwise stated) and stirred continuously to avoid settling. No beads were added in the case of control biofilms. This concentration was chosen based on our previous work, which demonstrated that AMR development was independent of MP concentration within the tested range. This concentration, This concentration, higher than typical wastewater MPs concentrations, allows for a clear mechanistic understanding of MP–biofilm interactions. Additionally, an elevated particle concentration was necessary for the glass beads (which are denser), to ensure sufficient attachment and biofilm colonization.

### 3.2 Antibiotic susceptibility tests

After growth, ciprofloxacin hydrochloride (MP Biomedicals, USA) was added to the media at varying concentrations between 50 and 350 μg/mL. Biofilms were then exposed to continuous flow with the antibiotic for 24h, at the same rate as used for growth. Then, propidium Iodide (PI) (Sigma-Aldrich, USA) was properly diluted and added to the flow cell per manufacturer's instructions. Stained biofilms were imaged with an inverted confocal laser scanning microscope (CLSM) (Olympus FV3000) with a 40 × objective. PI was used to observe non-viable (dead) cells in the biofilm, and GFP expression was used to visualize viable or total number of cells if channels were superimposed. Z-stacks with a step size of 0.5 μm were captured at four different locations to obtain a 3D visualization of biofilm viability, starting at the base of the biofilm (distance from the attachment surface = 0 μm) and finishing at the biofilm interface with the liquid media. Biofilm viability exposed to different concentrations of ciprofloxacin was then compared with a control without antibiotic in all cases.

Z-stacks were analyzed using FIJI (ImageJ, USA), following an adapted version of a previously published protocol and calculations [21]. Briefly, images were cleaned by removing background noise, after which a threshold and segmentation were applied. Pixel counts for both the green (GFP) and red (PI) channels were obtained to calculate the percentages of viable and non-viable bacteria. Cells showing overlap between GFP and PI signals were classified as non-viable.

### 3.3 Biofilm structure analysis

Optical Coherence Tomography (OCT) (Telesto® Series SD-OCT System, Thorlabs Inc., Germany) was used to obtain cross-sectional biofilm profiles at micrometer-scale spatial resolution. Images were used to calculate biofilm thickness, roughness, and overall structure. The system provided 5.5 μm axial resolution and 3.5 mm imaging depth in water.

Thickness was averaged from 10 to 12 points in each image, in at least five different sections in each flow cell, and repeating this procedure in two different replicates for each condition. Relative roughness coefficient, $R_{a}^{\prime }$, was calculated from the thickness values previously determined, $L_{f,i}$, and incorporating the biofilm thickness, $\underline{L_{f}}$, using the following equation:

Eq. [1] $$R_{a}^{\prime }=\frac{1}{N}\sum_{1}^{N}\left(\frac{\left|L_{f,i}-\underline{L_{f}}\right|}{\underline{L_{f}}}\right)$$

### 3.4 Biofilm mechanical properties

Microbead microrheology was used to calculate biofilm creep compliance. Biofilms were grown under flow under the same conditions previously described, but adding 100uL/L of 1 μm diameter polystyrene fluorescent particles (Fluoresbrite 18660, Polysciences). The media contained approximately 4.55 × 10^10^ particles/mL. 60-second videos were acquired using a CLSM (Olympus FV3000) at a 30-fps frame rate. At least 10 videos were recorded for each biofilm, at approximately 10 – 15 μm depth (considering the attachment surface to be Z = 0). Each video captured a minimum of 4 particles. Each experiment was repeated three times. The videos were analyzed using TrackMate in FIJI and MATLAB (MATLAB R2022a, Mathworks, Inc.), following existing procedures and parameters [20,22,23]. Mean square displacement (MSD) curves and mean MSD were calculated from microbead trajectories, and creep compliance J(t) was calculated from:

Eq. [2] $$J\left(t\right)=\frac{3\pi d}{4k_{B}T}MSD\left(t\right)$$

where J = creep compliance (Pa^−1^), d = particle diameter (m), $k_{B}$ = Boltzman constant (J*K^−1^), T = temperature (K), and t = time (s).

For effective creep compliance, J_eff_, the median value between 10 and 40 s was calculated as the most representative value. Each experiment was repeated at least twice for every condition, with a minimum of five technical replicates per experiment.

### 3.5 Characterization of EPS constituents

Polysaccharides, Proteins, and eDNA were considered the main EPS constituents of all biofilms. Their relative abundance was determined using CSLM and component-specific fluorescent stains, and the relative area of each fluorescent signal in different sections of the biofilms was quantified. All stains were freshly diluted from stocks and added with a peristaltic pump at the same rate as the flow rate used for growth, to avoid biofilm disturbance. Stains were added simultaneously in a total volume of 4 mL and allowed to flow through the biofilm completely. Table 1 shows the stains and the concentration used.

**Table 1:** Fluorescent dyes for EPS staining.

Stain	Concentration	Solvent	Ex/Em (nm)	Target
Wheat germ agglutinin-Alexa Fluor 405 plus	10 μg/mL	HBSS	405/450	Polysaccharides
Concanavalin A-Alexa Fluor 405 plus	100 μg/mL	0.1% NaHCO_3_		
Sypro Orange	1:5000	7.5% Acetic acid	300-370/570	Proteins
TOTO-1 Iodide	1:10000	TE buffer	514/533	eDNA

### 3.6 Statistical analysis

The significance between single-grouped variables was determined using an ordinary one-way analysis of variance (ANOVA). Before performing the ANOVA, the residuals were tested for normality using the Shapiro-Wilk test, confirming a Gaussian distribution. Each variable was then compared to the mean of a control, which varied depending on the study. Multiple comparisons were corrected with the Dunnett test, with P values adjusted accordingly. Finally, the residuals were tested for homogeneity of variances and potential clustering through the Brown-Forsythe and Bartlett's tests. The one-way ANOVA was chosen because it allows for comparing the means of variables across groups under the assumption of normally distributed residuals, which was confirmed here with the Shapiro-Wilk test. Dunnett's test was applied for post hoc comparisons, as it is well-suited for comparing each group to a control with minimized type I error. Finally, the Brown-Forsythe and Bartlett's tests were used to assess variance homogeneity, ensuring the robustness of the ANOVA results. For multivariable groups, two-way ANOVAs (Mixed Model) was used to determine significance. Here, the protocol follows the above steps, except, within a given row, each mean was compared to its respective column value. We corrected for multiple comparisons using statistical hypothesis testing (Turkey) and each P value is automatically adjusted to account for multiple comparisons. The family-wise alpha threshold and confidence levels was determined to be 0.05 (95% confidence interval). P-values below 0.0001, 0.001, 0.01, and 0.05 were denoted with ****, ***, **, and *, respectively.

## 4 Results

### 4.1 Antibiotic susceptibility

**Fig. 1:**
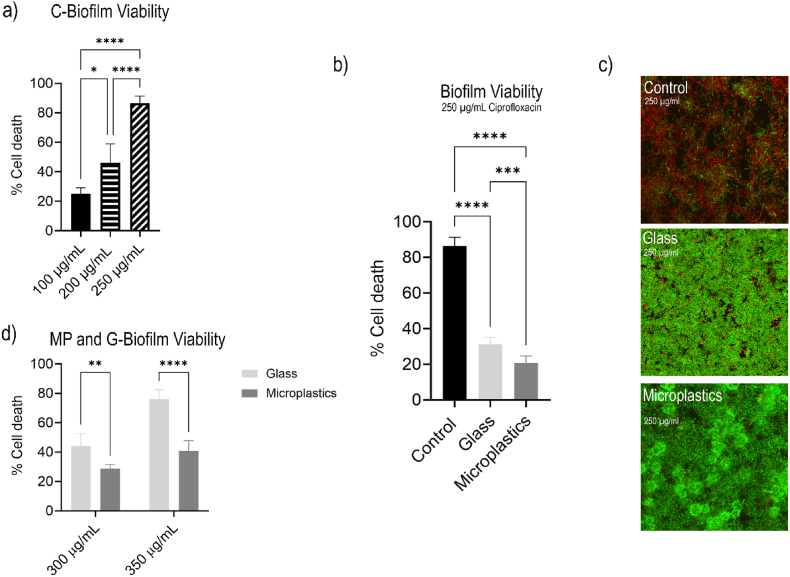
**Antibiotic susceptibility of biofilms exposed to ciprofloxacin for 24 h**. (a) C-biofilms at 100, 200, and 250 μg/mL, (b) C-, G-, and MP-biofilm viability at 250 μg/mL, (c) Respective CLSM projected z-stacks of biofilms. (d) MP- and G-biofilms at varying concentrations of 300 and 350 μg/mL of ciprofloxacin. Green fluorescence denotes live *E. coli* cells and red indicates dead cells from exposure to ciprofloxacin. (For interpretation of the references to colour in this figure legend, the reader is referred to the Web version of this article.)

We first tested the antibiotic susceptibility of control biofilms (i.e. without microbeads) across a range of ciprofloxacin concentrations. The concentrations required to reduce biofilm viability were substantially higher than the minimum inhibitory concentration (MIC) for planktonic, non-resistant *E. coli* [23]. After 24h exposure to 100 μg/mL ciprofloxacin, most of the biofilm remained viable (77.2 ± 4.1 %). Increasing the concentration to 200 μg/mL reduced viability to approximately 50 % (53.8 ± 12.1 %), and 250 μg/mL further decreased it to an average of 15.9 ± 4.9 % viable cells (Fig. 1a).

We then compared cell viability—determined as percentage of living cells compared to cells that died after antibiotic exposure—in MP-biofilms and G-biofilms, using a minimum ciprofloxacin concentration of 250 μg/mL, as lower concentrations did not show any appreciable effect in decreasing viability (Fig. 1c). In both cases, the antibiotic was noticeably less effective, with nearly 80 % of MP-biofilms and 70 % of G-biofilms remaining viable after treatment. The MP-biofilm exhibited a significant increase in biofilm viability compared to the G and C-biofilms (P = 0.0009, P < 0.0001, respectively). While biofilm viability decreased for both biofilms with increased concentrations of ciprofloxacin, approximately 60 % of the MP-biofilm still remained viable compared to the 24 % within the G-biofilm after exposure to 350 μg/mL for 24h (Fig. 1d, P < 0.0001).

**Fig. 2:**
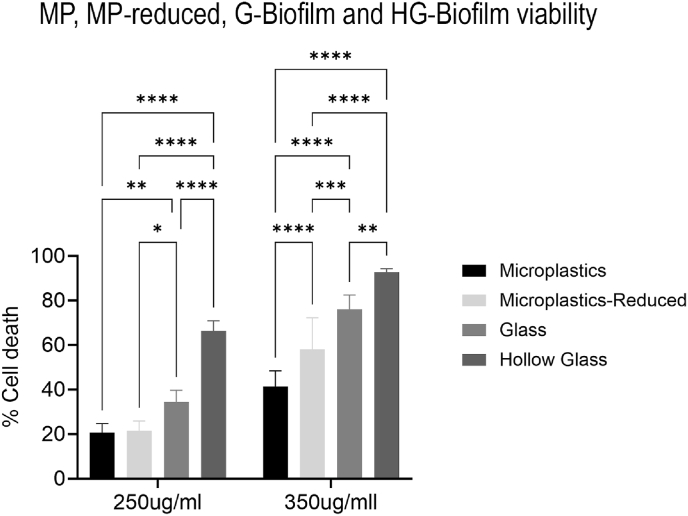
**Effect of MP concentration and weight/density factors on antibiotic susceptibility.** Varying concentrations of ciprofloxacin with different concentrations of microplastics (100 and 10 beads/μL) and different compositions of microbeads (polystyrene, glass, and hollow glass).

Following the initial characterization of antibiotic susceptibility to the varying types of biofilms (C, G and MP), the conditions of the variables were changed to elucidate if (1) concentration of MPs was a determining factor in higher resistance rates and (2) weight/density was a factor in the lower G-biofilm resistance. To do this, a ten-fold reduced concentration of microplastics (Microplastics-Reduced or MP-R-Biofilm) was grown and exposed to 250 and 350 μg/mL. In both cases, MP-R-Biofilm viability was significantly more viable than the G-biofilm, with 76.7 ± 6.9% and 42 ± 14.1% of cells remaining viable after 24h exposure (P = 0.0232 and P = 0.0002, respectively) and had no significant difference in viability from the MP-biofilm at 250 μg/mL (Fig. 2).

To understand the weight/density factor, hollow glass microbeads (HG-biofilm) were added to the media at the same concentration as the MP- and G-biofilms and the biofilms were grown using the same flow conditions. The HG-biofilm was then exposed to 250 and 350 μg/mL of ciprofloxacin for 24h. In both cases, the HG-biofilms had significantly less viability compared to all other conditions, at 33.6 ± 4.5 % and 7.3 ± 1.6 % cells remaining viable (P < 0.0001 for all conditions) (Fig. 2).

### 4.2 Biofilm structure

**Fig. 3:**
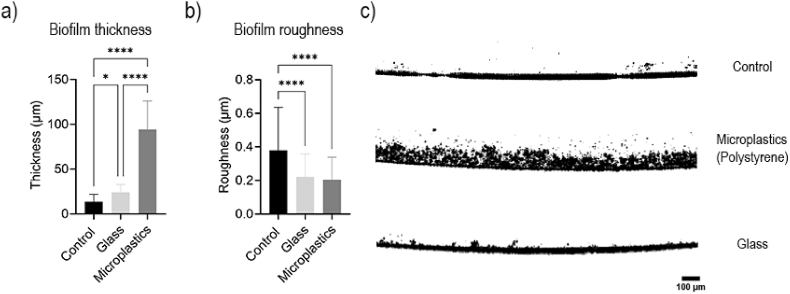
**Biofilm structure and growth patterns in the presence and absence of microbeads.** Biofilm (a) thickness, (b) roughness, and (c) structure of C-biofilm, G-biofilm and MP-biofilm.

To understand the differences in biofilm viability, we analyzed morphological changes in the structure of all biofilms, including MP-, G-, and C-biofilms. We explored growth profiles and analyzed changes in the structure, as well as thickness and roughness patterns, using Optical Coherence Tomography (OCT). Typical images from the OCT are shown in Fig. 3c. While C-biofilms had an average thickness of 13.71 ± 8.24 μm, G-biofilms were 75.7% thicker, with an average of 24.1 ± 3.1 μm (P = 0.0143). However, the MP-biofilms were significantly thicker than C- and G-biofilms, with an average thickness of 95.3 ± 22.9 μm (P < 0.0001) (Fig. 3a).

We also explored growth patterns and changes in biofilm morphology with different types of beads under the OCT (Fig. 3c). Regardless of the thickness, all biofilms appeared uneven, with patchy coverage. This was particularly evident in C- and G-biofilms, with C-biofilms showing the most irregular coverage (Fig. 3c). Quantitatively, C-biofilms exhibited a significantly higher roughness at 0.38 ± 0.31 μm compared to MP- and G-biofilms (0.25 ± 0.14 μm and 0.24 ± 0.14 μm, respectively, P < 0.0001) (Fig. 3b). The localized growth pattern in C-biofilms may reflect uneven colonization, possibly associated with the absence of beads. In addition, MP-biofilms exhibited dense spots, with darker, rounded regions, followed by spaces in between with lighter coverage and streamers, likely corresponding to localized biofilm growth surrounding the microplastics (Fig. S1). In OCT imaging, higher-contrast areas indicate greater signal intensity or optical reflectivity. Specifically in biofilms, this is associated with higher cell density, suggesting that MPs promote localized zones of dense biofilm formation.

**Fig. 4:**
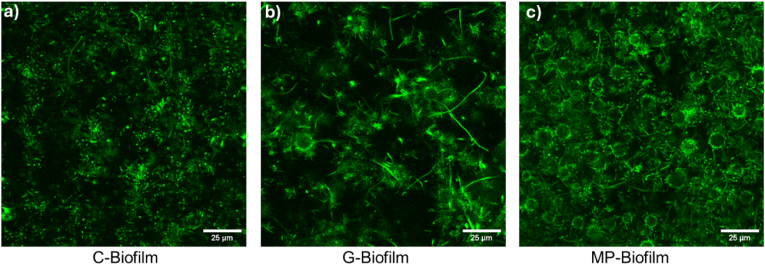
**Confocal cross-sectional images of biofilms at z=10 μm from the base (z=0).** Green fluorescence denotes *E. coli* GFP cells. (For interpretation of the references to colour in this figure legend, the reader is referred to the Web version of this article.)

Fig. 4 depicts the spatial distributions of microbeads using confocal microscopy to image G-biofilm and MP-biofilm, compared to the C-biofilm. In both microbead biofilm conditions—glass and microplastic—the same amount of microbeads was added to the media (approximately 1 × 10^6^ microbeads/mL). However, the number of beads embedded in the biofilm matrix was approximately 20-fold higher in the case of the MP-biofilm, compared to the G-biofilm.

### 4.3 Biofilm mechanical properties

**Fig. 5:**
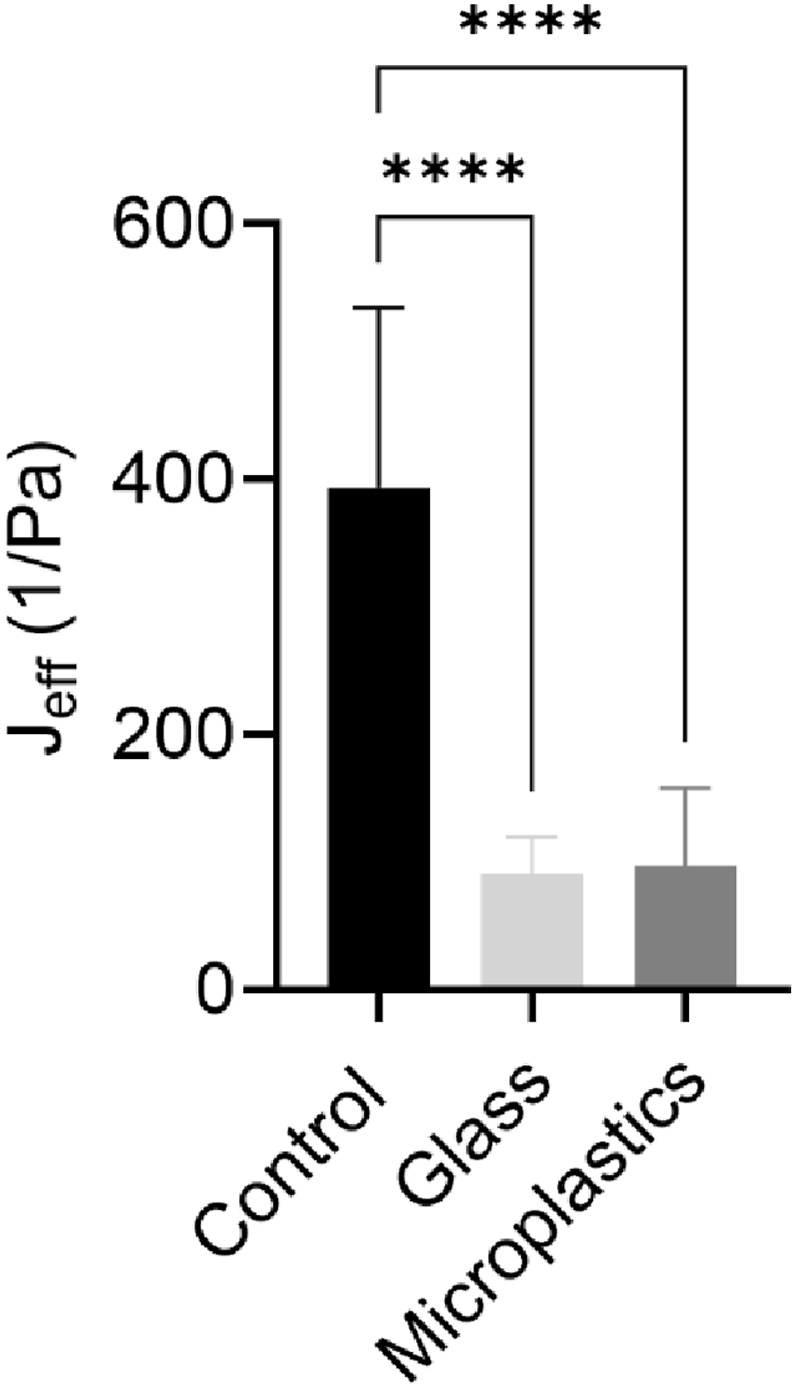
**Effective creep compliance for C-, MP-, and G-biofilms**.

Because increased antibiotic tolerance has been linked to greater biofilm stiffness, we characterized the mechanical properties of the three biofilm types using microrheology and creep compliance measurements [15]. All three biofilms exhibited viscoelastic behavior, as indicated by power-law fits to the creep compliance curves (Fig. S2). The G-biofilm displayed a diffusive exponent of approximately 0.25, consistent with predominantly elastic behavior. In contrast, the MP-biofilm and control biofilm showed higher exponents of ∼0.50, reflecting greater viscous contributions while still maintaining elasticity. Among the three, the control biofilm had the highest creep compliance (J_eff_ = 393.80 ± 140.30 1/Pa), suggesting it was the weakest and most deformable (Fig. 5). In contrast, both the G-biofilm (91.07 ± 28.54 1/Pa) and MP-biofilm (97.32 ± 61.00 1/Pa) exhibited significantly lower creep compliance values, indicating that the presence of microbeads—regardless of type—enhanced biofilm mechanical strength (Fig. 5, P < 0.0001). This mechanical reinforcement may help explain the increased antibiotic tolerance observed in both microbead-associated biofilms. However, since the G- and MP-biofilms had a similar stiffness, these data alone cannot account for the higher antibiotic tolerance observed in the MP-biofilm, suggesting that additional factors, such as differences in EPS composition, cell–surface interactions, or microplastic-specific physicochemical effects, may play a role.

The measurements of biofilm mechanical properties showed some correlation with biofilm structure and growth patterns (Fig. 3C). Biofilms are inherently heterogenous. Particularly, due to strong adhesiveness of cells to MPs, dense and highly cohesive biofilm structures developed immediately surrounding the microplastics and, exhibiting localized dense growth surrounding the MPs and more porous, streamer-like farther from the microbeads. This heterogeneity likely contributed to the higher variability, reflected in the large standard deviations observed in their creep compliance measurements.

### 4.4 Biofilm EPS characteristics

**Fig. 6:**
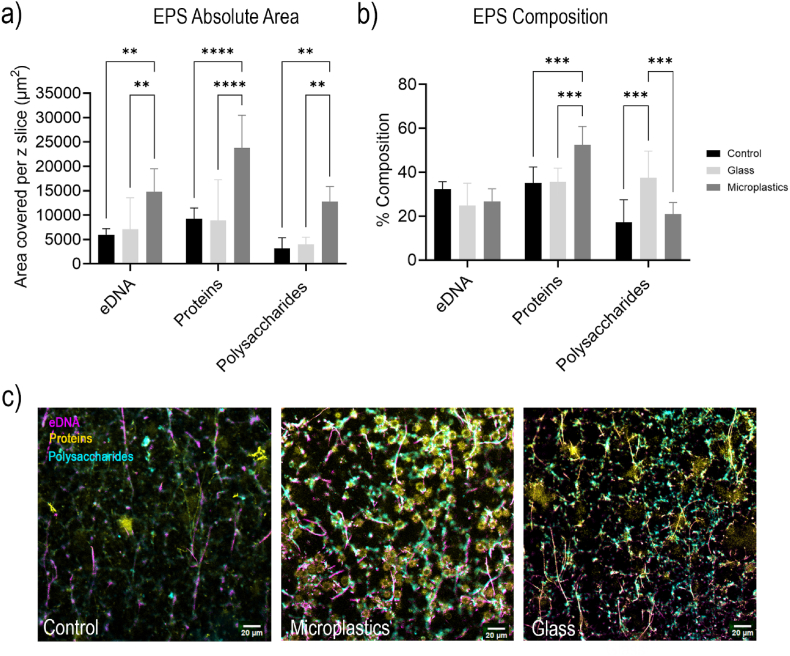
**EPS composition and distribution in C-, MP- and G-biofilms** (a) Total area of EPS signal in a cross-sectional CLSM capture (101250 μm^2^), (b) percent composition of each constituent (eDNA, proteins, polysaccharides) found in EPS, (c) CLSM cross-sectional images of biofilms at the midpoint of the biofilm from the base (z = 0). Magenta, yellow, and cyan denote eDNA, proteins, and polysaccharides, respectively. (For interpretation of the references to colour in this figure legend, the reader is referred to the Web version of this article.)

Quantification of EPS components revealed a significant increase in absolute coverage of extracellular DNA (eDNA), proteins, and polysaccharides in MP-biofilms compared to the G- and C-biofilms (Fig. 6a, P < 0.01 for eDNA and Polysaccharides and P < 0.0001 for Proteins). MP-biofilms promoted significantly higher total EPS accumulation for all constituents, with a 2.5-fold eDNA, 2.6-fold proteins, and 4-fold polysaccharides total area increase, compared to the C- and G-biofilms. Notably, proteins were the predominant component across all conditions, comprising about 23% ± 6% of the MP-biofilm.

When normalized to total EPS, substrate-associated biofilms displayed marked differences in composition (Fig. 6b). The largest increase observed was for proteins, which represented a 1.5-fold larger fraction of the EPS matrix in the MP-biofilm (53 ± 8 %) compared to G- and C-biofilms (P < 0.0001 (MP-C), P < 0.01 ((MP-G), P < 0.05 (G-C)). Notably, polysaccharides were found to have been enriched in G-biofilms with a relative abundance of 37.5 ± 11.2 % in the EPS. This represents a 1.8-fold increase relative to the C-biofilms (P < 0.05) and a 1.5-fold increase relative to the MP-biofilm (P < 0.05), while eDNA contributions remained relatively consistent across treatments.

Representative fluorescent micrographs confirmed these quantitative trends, showing denser and more extensive EPS networks in biofilms grown on microplastic and glass substrates relative to controls (Fig. 6c). The micrographs reveal abundant protein (yellow) and polysaccharide (cyan) signals intertwined with eDNA (magenta), forming more complex and entangled architectures on the microbeads—independently of the material—compared to the sparse networks in controls without microbeads.

## 5 Discussion

In this study, we compared the antibiotic susceptibility of *E. coli* biofilms grown under flow in the presence of microplastics, glass microbeads, or control biofilms without beads. We further analyzed structural changes, mechanical stability, and EPS composition in each biofilm type to identify possible mechanisms responsible for the enhanced resistance.

Our findings illustrate that the presence and type of abiotic surfaces—particularly microplastics and glass microbeads—strongly influence biofilm antibiotic susceptibility, structural characteristics, and EPS production. Biofilms formed in association with microbeads (MP-biofilm and G-biofilms) displayed significantly greater resistance, with MP-biofilms showing the highest tolerance across all tested concentrations (as shown in Fig. 1b–d). Furthermore, reducing the inlet concentration of MPs to one tenth of the initial concentration used for both MP- and G-biofilms continued to show significantly higher tolerance to ciprofloxacin than G-biofilms (Fig. 2). This was accompanied by a four-fold increase in microbead uptake and overall larger biofilm thickness. This suggests that the presence of MPs intensifies biofilm antibiotic tolerance beyond what is typically expected from surface-mediated growth alone.

Physiological stratification is commonly driven by gradients in nutrients availability, resulting in reduced metabolic activity in deeper portions of the biofilm due to limited nutrient concentration and diffusion. This effect is particularly stressed in thick biofilms. These metabolically less active cells are known to be less susceptible to many antibiotics, including ciprofloxacin. In our study, MP-biofilms were considerably thicker than the control biofilms without microbeads. However, in our results, we observed that variation in susceptibility primarily related to proximity to MPs, rather than associated with biofilm depth and metabolism; cells in close contact with or immediately attached to MPs remained predominantly viable, while cells located further or in between the beads exhibited increased susceptibility, as indicated by a higher proportion of dead cells. This spatial pattern was observed in both outer and inner regions of the biofilm. These results suggest that, under our experimental conditions, MPs-associated effects play a more prominent role in shaping antibiotic tolerance than depth-dependent physiological stratification.

Structural analyses provide mechanistic insight into this phenomenon. OCT revealed that MP-biofilms were substantially thicker than both C- and G-biofilms, with an architecture characterized by localized dense zones surrounding microplastic particles (Fig. 3a–c). CLSM images confirmed that microplastics were more efficiently incorporated into the biofilm matrix compared to glass beads, which remained sparsely embedded (Fig. 4). This difference in microbead incorporation may explain the formation of thicker and denser MP-biofilms, as the surface chemistry and hydrophobicity of MPs likely promote stronger cell–surface interactions [12]. The spatial heterogeneity observed in MP-biofilms—dense clusters interspersed with streamer-like branches (Fig. S1)—further indicates localized growth patterns with high cell density, which likely facilitate cell-to-cell communication. This can potentially facilitate gene exchange and adaptative mutations, promoting antibiotic resistance, as suggested in previous studies [19,20]. However, the potential role of increased gene transfer and mutations in enhancing antibiotic tolerance in MP-biofilms needs to be directly evaluated in future studies, likely through detailed sequencing-based comparisons with control biofilms grown in the absence of microbeads. Prior literature has also shown that MPs can adsorb and accumulate genetic material, providing not only a favorable attachment surface for microorganisms, but also additional substrate for gene exchange [[24], [25], [26]]. This is particularly relevant in aquatic environments, especially within wastewater systems, where the coexistence of MPs, antibiotic traces, and antibiotic resistance genes (ARGs) can accelerate the formation of highly tolerant biofilms. Consequently, the densely grown biofilms in wastewater systems attached to MPs likely have greater access to adsorbed ARGs and horizontal gene transfer rates, ultimately contributing to the already ongoing critical AMR development globally.

Mechanical characterization showed that both MP- and G-biofilms exhibited significantly lower creep compliance than controls, indicating increased stiffness and resistance to deformation (Fig. 5). This higher mechanical strength has been previously linked to enhanced antibiotic tolerance in biofilms [20]. However, despite the similar stiffness between MP- and G-biofilms, the differences in antibiotic susceptibility indicate that biofilm mechanics alone cannot fully explain the superior antibiotic tolerance of MP-biofilms. Instead, EPS composition emerges as a likely determinant.

EPS quantification showed that MP-biofilms had significantly higher accumulation of all major components—proteins, polysaccharides, and eDNA—with proteins particularly enriched relative to both C- and G-biofilms (Fig. 6a). Given that proteins in the EPS are known to enhance biofilm stability, increase nutrient retention, and sequester antimicrobials, the disproportionately protein-rich EPS matrix of MP-biofilms may provide a mode that synergizes with increased thickness and density to reduce antibiotic efficacy [27]. While *E. coli* biofilms are known to have higher protein content, the highest value of proteins was MP-specific, with the G- and C-biofilms producing a significantly lower percentage of protein content (Fig. 6b). This suggests that the material properties of MPs allow for a higher attachment and likely growth rates, followed by greater rates of EPS production. In the environment, MPs have been found in aquatic and host-associated systems to quickly acquire a “conditioning film” of organic matter and proteins [28,29]. Within this niche, bacteria such as *E. coli* have been shown to become better biofilm formers, changing their phenotypes to attach and proliferate on surfaces faster [30]. These factors will ultimately enrich proteinaceous components in the biofilm matrix relative to glass or control. This higher protein content within the EPS matrix, together with the enhanced antibiotic tolerance, may be partially attributable to the presence of antibiotic-modifying enzymes, such as lyases, hydrolases and redox enzymes within the matrix [31,32]. These proteins are known to modify or inactivate antibiotics by cleaving chemical bonds or restricting biding sites, reducing their efficiency, and therefore enhancing tolerance to antibiotics. However, the specific identities and activities of the proteins within MP-biofilms were not directly assessed in this study and remain to be explored in future work.

Combining our findings on EPS composition and spatial distribution with biofilm structural analysis, we found that the localized zones of high cell density in MP-biofilms correlated with enhanced overall EPS production, likely providing increased protection in these regions and contributing to the overall elevated antibiotic tolerance, a mechanism also reported in previous literature [23]. It is important to note that although EPS composition offers a major distinction between MP-, G- and Control biofilms, biofilm stiffness is generally linked to EPS composition, and has been associated with enhanced antibiotic tolerance [33,34]. In this line, previous studies using mutants deficient in specific EPS components have demonstrated increased antibiotic susceptibility and reduced biofilm stiffness or impaired biofilm formation [35]. Building on these findings, future studies could assess antibiotic susceptibility in *E. coli* biofilms mutants lacking major EPS-associated proteins in the presence of MPs to better understand whether the enhanced tolerance is driven by protein components of the EPS matrix.

While MP-biofilms had the highest level of resistance to ciprofloxacin, G-biofilms were still found to be more resistant than C-biofilms (Fig. 1c). Glass provides fewer chemical anchoring sites than microplastics due to its hydrophilic and smooth surface. Previous research has shown that biofilm-forming organisms on glass often compensate by producing greater amounts of polysaccharides, which serve as structural scaffolds and enhance adhesion on strong, non-porous materials [30]. Thus, the higher proportion of polysaccharides in the G-biofilms reflects biofilm architecture optimized for mechanical stability rather than rapid bacterial proliferation (in which microplastics are a unique material to allow this) (Fig. 6b).

Our results quantitatively and mechanistically extend the now-well-established idea of a plastic-associated “plastisphere” by showing, in a controlled flow system and a single species, that MPs do more than simply host biofilms—they actively modulate biofilm physiology in several ways that significantly raise antibiotic tolerance. Prior work has shown that MPs rapidly acquire conditioning films of proteins/organic matter that alter surface chemistry and promote colonization relative to inert hydrophilic glass, and that hydrophobic materials favor stronger cell–surface interactions and thicker biofilms *on* the surface [[28], [29], [30],^36^]. However, the mechanistic understanding of biofilm-based antibiotic tolerance pertaining to other realistic environmental conditions—such as biofilms that might incorporate MPs rather than the other way around—is noticeably lacking. Within the biofilm complex, our observations of greater MP uptake, thicker architecture, and localized dense zones are consistent with—and mechanistically grounded in—surface-chemistry literature previously focused on planktonic bacteria research [30,37]. We also add that larger, protein-enriched EPS appears to be a distinguishing feature of biofilms with embedded MPs, which could be a major factor in the development of a stiffer structure and decreased susceptibility to antibiotics.

If the presence of MPs systematically produces larger biofilm physiologies that favor protein-rich, mechanically robust, and gene-exchange-prone states, then MPs in any of the environments they have infiltrated may act as reservoirs of drug-tolerant communities and antibiotic-resistant genes (ARGs). This conclusion aligns with recent experimental and *meta*-omics evidence that MP surfaces elevate ARG abundance and transfer, and with emerging reports that MP exposure can accelerate significant multidrug resistance development in *E. coli* [28,38]. From a control standpoint, this suggests that material choice (surface chemistry/hydrophobicity) and EPS-targeted strategies (enzymatic or chemical disruption of proteins/eDNA) should be considered in environmental management with wastewater treatment as a primary target.

The observations found in this study set critical implications when translated into a broader environmental perspective. As these robust and highly tolerant MP-biofilms develop, they can serve as vectors of AMR to the wider environment. Furthermore, the stability and interactions between MPs and biofilms developed in these environments may ultimately alter local microbial ecology and biogeochemical cycling. It is important to acknowledge that our study was limited to single bacterial species and only one type of MPs, building upon results from previous findings. Extending these experiments to include biofilm growth of diverse environmental species and with a broader range of MP types are necessary to fully understand how these factors influence biofilm structure and antimicrobial tolerance. In addition, future studies should address the potential effects of MP degradation and additive leaching, as well as the development of resistant biofilm phenotypes, and changes in biofilm microbial communities in environmental samples. This highlights the need to view MPs not only as physical contaminants and active modulators of biofilm characteristics, but also to further study MPs and biofilm interactions for important ecological impacts in aquatic environments.

## 6 Conclusions

The results in this study suggest that MPs act not only as passive scaffolds but also as active modulators of biofilm physiology, promoting structural and biochemical traits that enhance antibiotic tolerance. Collectively, these data highlight the multifactorial basis of antibiotic tolerance in microplastic-associated biofilm communities. Enhanced thickness, localized dense growth, enhanced mechanical strength, and altered EPS composition converge to create highly resilient biofilms that withstand high concentrations of ciprofloxacin. Furthermore, even reduced concentrations of MPs (MP-R-biofilms) retain enhanced antibiotic tolerance relative to G-biofilms, stressing the power of MP surfaces in promoting resilience against antibiotic treatments. Moreover, the poor incorporation of glass microbeads and the enhanced susceptibility of hollow glass biofilms—less heavy and with the capacity to stay in suspension—suggest that density, surface chemistry, and hydrophobicity of microbeads all contribute to shaping biofilm resistance phenotypes. This raises important ecological and clinical concerns: microplastics in wastewater-like environments may serve as hotspots for persistent, drug-tolerant microbial communities, and further affect other aquatic environments. Understanding these interactions is essential for assessing the role of environmental MPs in driving AMR and for developing strategies to mitigate their impact in environmental settings.

## CRediT authorship contribution statement

**Yanina Nahum:** Writing – review & editing, Writing – original draft, Methodology, Investigation, Formal analysis, Conceptualization. **Neila Gross:** Writing – review & editing, Writing – original draft, Methodology, Investigation, Formal analysis, Conceptualization. **Johnathan Muhvich:** Writing – review & editing, Investigation, Conceptualization. **Muhammad H. Zaman:** Writing – review & editing, Supervision, Resources, Project administration, Conceptualization.

## Declaration of competing interest

The authors declare that they have no known competing financial interests or personal relationships that could have appeared to influence the work reported in this paper.

## Data Availability

Data will be made available on request.
