# Supplementary material for: Microplastics as active modulators of Escherichia coli biofilm characteristics and their implications on the development of antimicrobial resistance
Source: Biofilm. 2026 Feb 11;11:100355. doi: 10.1016/j.bioflm.2026.100355 (PMC12914106; doi:10.1016/j.bioflm.2026.100355)
Supplement: Multimedia component 1 [file mmc1.docx]

**Supplementary Information**

**Assessing the Impact of Microplastics on *Escherichia coli* Biofilm Characteristics and Their Implications on the Development of Antimicrobial Resistance**

Yanina Nahum*****^,1,2,3^, Neila Gross*****^,4^, Johnathan Muhvich^1^, Muhammad H. Zaman^1,2^

***These authors contributed equally to this work**

^1^ Department of Biomedical Engineering, Boston University, Boston, MA, USA

^2^ Center on Forced Displacement, Boston University, Boston, MA, USA

^3^ Odum School of Ecology, University of Georgia, Athens, GA, USA

^4^ Department of Materials Science and Engineering, Boston University, Boston, Massachusetts, USA

**
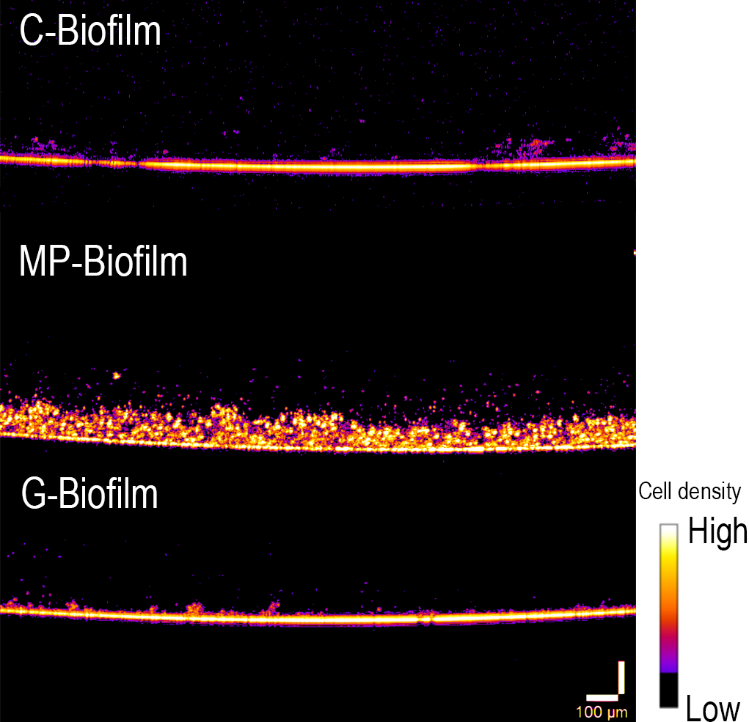
**

***Figure S1.*** ***Biofilm structure in the presence and absence of microbeads using an intensity gradient.*** *Control (top), Microplastics (middle), and Glass (bottom).*


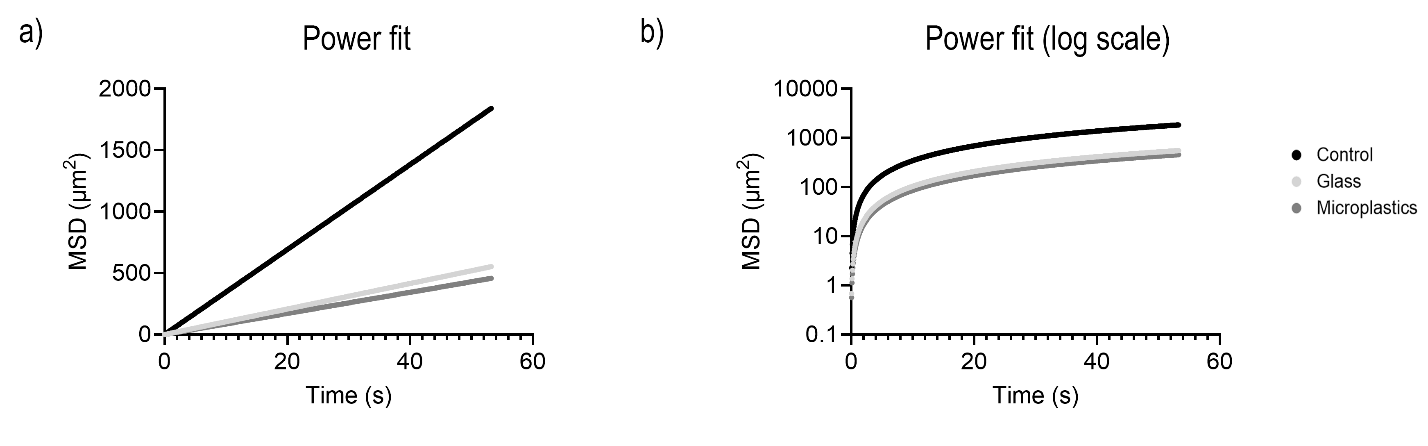


***Figure S2. Power law fits to creep compliance curves.*** *Linear (left) and log (base 10) scale (right).*
